# Adaptation of Microbial Communities to Environmental Arsenic and Selection of Arsenite-Oxidizing Bacteria From Contaminated Groundwaters

**DOI:** 10.3389/fmicb.2021.634025

**Published:** 2021-03-19

**Authors:** Sarah Zecchin, Simona Crognale, Patrizia Zaccheo, Stefano Fazi, Stefano Amalfitano, Barbara Casentini, Matteo Callegari, Raffaella Zanchi, Gian Attilio Sacchi, Simona Rossetti, Lucia Cavalca

**Affiliations:** ^1^Dipartimento di Scienze per gli Alimenti, la Nutrizione e l’Ambiente (DeFENS), Università degli Studi di Milano, Milano, Italy; ^2^Water Research Institute, National Research Council of Italy (IRSA-CNR), Rome, Italy; ^3^Dipartimento di Scienze Agrarie e Ambientali—Produzione, Territorio, Agroenergia (DiSAA), Università degli Studi di Milano, Milano, Italy

**Keywords:** arsenic, groundwater, arsenic dissolution, arsenite-oxidizing bacteria, *Pseudomonas* spp.

## Abstract

Arsenic mobilization in groundwater systems is driven by a variety of functionally diverse microorganisms and complex interconnections between different physicochemical factors. In order to unravel this great ecosystem complexity, groundwaters with varying background concentrations and speciation of arsenic were considered in the Po Plain (Northern Italy), one of the most populated areas in Europe affected by metalloid contamination. High-throughput Illumina 16S rRNA gene sequencing, CARD-FISH and enrichment of arsenic-transforming consortia showed that among the analyzed groundwaters, diverse microbial communities were present, both in terms of diversity and functionality. Oxidized inorganic arsenic [arsenite, As(III)] was the main driver that shaped each community. Several uncharacterized members of the genus *Pseudomonas*, putatively involved in metalloid transformation, were revealed *in situ* in the most contaminated samples. With a cultivation approach, arsenic metabolisms potentially active at the site were evidenced. In chemolithoautotrophic conditions, As(III) oxidation rate linearly correlated to As(III) concentration measured at the parental sites, suggesting that local As(III) concentration was a relevant factor that selected for As(III)-oxidizing bacterial populations. In view of the exploitation of these As(III)-oxidizing consortia in biotechnology-based arsenic bioremediation actions, these results suggest that contaminated aquifers in Northern Italy host unexplored microbial populations that provide essential ecosystem services.

## Introduction

Global freshwater is the main source of drinking water due to its natural supply stability and good microbial quality, and mainly relies on groundwater resources (Kim et al., 2011). Consumption of arsenic contaminated water is the main cause of poisoning for humans (Abernathy et al., 2003), leading to serious concerns in different countries worldwide, like China (Wang et al., 2019), India (Chakraborty et al., 2015), America (Welch et al., 2000; Bundschuh et al., 2012), South Eastern Asia (Mukherjee et al., 2006; Guo et al., 2014), and Europe (Katsoyiannis et al., 2014). The use of arsenic-contaminated water for human consumption or agricultural purposes causes several diseases to millions of people who are affected by poisoning disorders, such as dermatitis and cancer (Naujokas et al., 2013; Singh et al., 2015; Podgorski and Berg, 2020). Therefore, this emerges as a relevant topic, especially in developing countries where drinking water treatment processes are largely insufficient or rarely carried out (Pokhrel et al., 2009).

Arsenite [As(III)] and arsenate [As(V)] are the most frequently encountered arsenic inorganic species in groundwater, with As(III) being dominant in sedimentary aquifers due to sub-neutral pH and reductive conditions (Herath et al., 2016). Methylated species might also be present, although they are much more represented in organic carbon-rich environments such as agricultural or surface waters rather than oligotrophic environments like groundwaters (Hasegawa et al., 2010; Escudero et al., 2013). Although groundwaters are considered anoxic deep environments, aerobic microbial hotspots have been retrieved in subsurface aquifers all over the world (Castelle et al., 2013; Long et al., 2016; Bochet et al., 2020). Previous studies evidenced that deep microbial hotspots may substantially influence microbial communities, including both aerobic As(V)-reducing and As(III)-oxidizing bacteria, and their effect on Earth’s biogeochemical cycles that rely on soluble arsenic species (Cavalca et al., 2019). The reduction of As(V) mediated by intracellular arsenate reductase ArsC is a detoxification reaction that reduces As(V), which enters into the cell by phosphate antiporter Pst, to As(III). This is recognized by the efflux pump ArsB that extrudes arsenic out of the cell. Anaerobic reduction of As(V) mediated by the respiratory As(V) reductase allows several anaerobic microorganisms such as *Shewanella* sp. strain ANA3 to use As(V) as a terminal electron acceptor in the energy metabolism (Murphy and Saltikov, 2009). The oxidation of As(III) is both a detoxification strategy (i.e., mainly for heterotrophic microorganisms) and an energy gaining metabolism (i.e., mainly for chemolithoautotrophic bacteria). This reaction is carried out by arsenite oxidase AioA onto the cell membrane. *Pseudomonas arsenitoxidans* strain NT-26 is one of the best characterized chemolithoautotrophic strain (Ilialetdinov and Abdrashitova, 1981). Anaerobic As(III) oxidation mediated by arsenite oxidase Arx was primarily described in the halokaliphilic, arsenite-oxidizing bacterium *Alkalilimnicola ehrlichii* strain MLHE-1 (Zargar et al., 2010). Recently, Arx has also been characterized in the nitrate (NO_3_^–^)-respiring *Azoarcus* sp. CIB, a facultative anaerobic betaproteobacterium, able to resist to arsenic oxyanions under both aerobic and anaerobic conditions, and to use As(III) as an extra-energy source for anaerobic cell growth (Durante-Rodríguez et al., 2019).

Arsenic biogeochemistry is strictly related to microbial evolution on Earth since arsenic bacterial metabolisms have shaped the lithosphere (Zhu et al., 2014). Particularly, in arsenic-affected sedimentary aquifers, ferric iron [Fe(III)], As(V), and to a lesser extent manganese dissimilative reductions (i.e., anaerobic respiration) are implied in arsenic dissolution (Oremland and Stolz, 2005). Sedimentary history and the presence of peat deposits of different geological origins are important factors affecting microbial reactions (Rotiroti et al., 2015). In Holocene sedimentary deposits in China, Bangladesh, and South America, characterized by fresh and highly reactive organic compounds, arsenic rocks (i.e., arsenopyrite and arsenic-bearing amorphous iron (hydr)oxides) may undergo biological reductive dissolution, mediated by anaerobic respiration of organic compounds with As(V), Fe(III), or Mn(IV) as electron acceptors (Herath et al., 2016). In Europe, the Alpine sediments of Po Plain (Northern Italy) include one of the major water stocks (Agenzia Regionale per la Protezione dell’Ambiente (ARPA), 2018). Similarly to other deep aquifers, arsenic in the Po Plain area often exceeds the WHO limit of 10 μg L^–1^ (World Health Organization [WHO], 2001), due to low redox potential and oxygen levels, that promote the dissolution of arsenic bound to iron and manganese minerals. Since drinking water supply in this area relies heavily on groundwater wells, arsenic contamination represents a major health issue for the local population.

Whereas the microbial communities inhabiting arsenic-contaminated Holocene and Pleistocene aquifers in Asia have been widely characterized in the last years, limited information is available on the microbial communities that drive arsenic dissolution in contaminated groundwaters in Northern Italy (Cavalca et al., 2019). This work aims at deciphering the phylogenetic structure and role of microbial communities in relation to physicochemical characteristics in Italian arsenic-contaminated aquifer systems, and at demonstrating bacterial metabolic potential toward different arsenic species in view of their possible exploitation in water biological treatments.

## Materials and Methods

### Study Sites and Groundwater Sampling

Groundwater samples were collected from six public supply-wells located in the provinces of Varese (VA), Lodi (LO), Cremona (CR1 and CR2), Brescia (BS), and Mantova (MN) (Lombardia, Northern Italy) (Table 1 and Supplementary Figure 1). The sampling sites are distributed over the Alpine Pleistocene sediments of Po Plain, including aquifers with different depth within a multilayer system characterized by vertical alternations of sands (aquifer units) and silty clays (aquitard units) (Ori, 1993). The sites were chosen from the dataset of the Regional Agency for Health Prevention and Environmental Protection (ARPA) of Lombardy on the basis of different arsenic concentration in groundwaters, all above 10 μg L^–1^, which is the established drinking water limit by drinking water Directive 98/83/EC (Council Directive, 1998), implemented in Italy by D. Lgs. 31/2001. Before sampling, groundwater was flushed out from wells until temperature, pH, dissolved oxygen, and redox potential (Eh) were stabilized. Groundwater samples were collected into acid-washed and autoclaved 25 L polyethylene containers. At each site, 25–100 L of water were collected and transferred to the laboratory at 4°C in the dark.

**TABLE 1:** Site location and characteristics.

Sample ID	Location	Coordinates	Type	Use	Depth (m)
LO	Brembio	N 45°13′24.283″ E 9°34′14.739″	Well	Livestock	22
CR1	Paderno Ponchielli	N 45°14′41.474″ E 9°56′54.589″	Piezometer	Irrigation	24
MN	Redondesco	N 45°10′29.384″ E 10°30′35.891″	Public well	Potable	182
VA	Somma Lombardo	N 45°40′55.6578″ E 8°38′43.303″	Public well	Potable	92
BS	Pontevico	N 45°16′50.192″ E 10°5′32.603″	Well	Irrigation	50
CR2	Pescarolo ed Uniti	N 45°11′37.334 E 10°11′24.918″	Public well	Potable	210

*All provinces were situated in Lombardia region (Northern Italy).*

### Physicochemical Characterization of Groundwater Samples

Physicochemical parameters, such as temperature (T), redox potential (Eh), dissolved oxygen (DO), pH, and electrical conductivity (EC), were measured *in situ* with Eh and pH multi-probe E PCE-228 (PCE Deutschland GmbH, Germany), DO-meter-HI 9146 (Hanna Instrument US Inc., Woonsocket, United States, and WTW 340i Ec-meter (VWR International, Wayne, PA).

Measurements of total, inorganic [As(III), As(V)] and methylated arsenic species were performed by high-performance liquid chromatography (HPLC) on an anion exchange column PRP-X100 (250 × 4.6 mm, 5 μm) fitted with a pre-column and coupled with Inductively Coupled Plasma Mass Spectrometer (Bruker AURORA M90 ICP–MS, Bruker). For this analysis, non-acidified groundwater samples were passed through acetate cellulose 0.22 μm filters, immediately after sampling. Iron species were determined spectrophotometrically immediately after sampling, according to the APHA-AWWA-WEF standard ortophenathroline method (num. 3500-Fe IRON, American Public Health Association (APHA) et al., 2012).

The determination of dissolved ions (Fe, Mn, P, Ca, K, Mg, Na) was conducted by ICP-MS on filtered (pore diameter 0.22 μm) groundwater samples collected in 50 mL tubes and stored at −20°C. All samples were acidified to 2% HNO_3_ (v/v) and added with proper internal standards [Scandium (^45^Sc), Yttrium (^89^Y), Terbium (^159^Tb)] with concentrations ranging from 0 to 1 mg L^–1^ and prepared from a multi-standard solution (Agilent Technologies, United States). Nitrate, ammonium and sulfate were measured spectrophotometrically using NANOCOLOR^®^ kits (Test-064, Test-004, Test-086, respectively, Macherey-Nagel GmbH & Co., Germany), according to the manufacturer’s instructions. Sulfides in groundwater samples were analyzed spectrophotometrically according to the Cline method (Cline, 1969). Dissolved organic carbon (DOC) was determined in accordance with the International Organization for Standardization (ISO) 15705 (2002) method for the determination of COD (Chemical Oxygen Demand). This method exploits organic matter oxidation with potassium dichromate/sulfuric acid at 150°C during a period of 2 h (Test-027 Nanocolor^®^, Macherey-Nagel). As the last step, final dichromate concentration is detected by a filter photometer (PF-12 Macherey-Nagel). The results, expressed by the photometer as mg O_2_⋅L^–1^ of water extract, were recalculated as Dissolved Carbon mg DC⋅g^–1^ OM (milligrams of dissolved carbon per gram of organic matter).

### Fluorescent Cell Staining Methods

Each water sample was fixed in formaldehyde solution (FA, 1% v/v final concentration) and kept at 4°C for a maximum of 24 h. The volumetric absolute cell counting was carried out on fixed samples stained with SYBR Green I (1:10,000 dilution; Molecular Probes, Invitrogen) on a flow cytometer A50-micro (Apogee Flow System, Hertfordshire, England), equipped with a solid-state laser set at 20 mV and tuned to an excitation wave length of 488 nm. Apogee Histogram Software (v89.0) was used to plot and analyze the data; the light scattering signals (forward and side scatters) and the green fluorescence (530/30 nm) were considered for single cell characterization. Thresholding was set on the green channel and voltages were adjusted to place the background and instrumental noise below the first decade of green fluorescence. Samples were run at low flow rates to keep the number of events below 1,000 events s^–1^. The intensity of green fluorescence emitted by SYBR-positive cells allowed for the discrimination among cell groups exhibiting two different nucleic acid content (cells with Low Nucleic Acid content–LNA; cells with High Nucleic Acid content–HNA; Amalfitano et al., 2014).

An aliquot of fixed samples (100–300 mL depending on total cell abundance) was filtered on polycarbonate membrane filters (pore size 0.2 mm, 47 mm diameter, Millipore, MA, United States) by gentle vacuum (<0.2 bar) and washed with 20 mL of Milli-Q water. The filters were stored in Petri dishes at −20°C until further processing. Fluorescence *in situ* hybridization combined with catalyzed reporter deposition (CARD-FISH) was performed following the protocol optimized by Fazi et al. (2007, 2013). The following rRNA-target HRP-labeled probes (Biomers, Ulm, Germany) were used: EUB338 I-III for Bacteria, ALF968 for *Alphaproteobacteria*, BET42a for *Betaproteobacteria*, GAM42a for *Gammaproteobacteria*, DELTA495 for *Deltaproteobacteria*, CFX and GNSB for *Chloroflexi*, LGC354mix for *Firmicutes*, CF319a for *Flavobacteria*, PLA46 for *Planctomycetes*, TM7905 for TM7, HGC69A for *Actinobacteria* and ARCH915 for Archaea. Details of probes are available at probeBase (Greuter et al., 2016). The stained filter sections were inspected on a Leica DM LB30 epifluorescence microscope (Leica Microsystems GmbH, Wetzlar, Germany) at 1000X magnification. At least 300 cells were counted in > 10 microscopic fields randomly selected across the filter sections.

### DNA Isolation

For the molecular analyses of the microbial communities, 25 L of groundwater were filtered for samples LO, CR1, MN, and VA, whereas 80–100 L of groundwater were filtered for samples BS and CR2. Water samples were filtered under controlled flow over 0.22 μm sterile hollow fiber filters (Mediakap-5, SpectrumLabs, United States), and these filters were stored at −20°C. All silicone pipes and connectors used in the filtering apparatus were sterilized by washing with 2.5% sodium hypochlorite and autoclaved. To detach the biomass from the filters, these were thoroughly rinsed with 10 mL of 0.2% (w/v) sodium pyrophosphate. The suspensions were centrifuged at 10,000 rpm for 25 min at 10°C. Total DNA was isolated from the pellets using the PowerSoil^®^ DNA Isolation Kit (Mo Bio Laboratories, Inc., Carlsbad, CA, United States), with two additional lysing steps at 65°C for 30 min and 90°C for 5 min. The quality of DNA was tested via agarose gel electrophoresis and the concentrations were determined with a ND-1000 spectrophotometer (Nanodrop Inc., Wilmington, DE).

### Illumina MiSeq 16S rRNA Gene Sequencing

Sequencing of bacterial 16S rRNA genes was performed in triplicate with primer pair 341F (5′—CCTACGGGAG GCAGCAG—3′) and 806R (5′–GGACTACHVGGGTWT CTAAT—3) (Takahashi et al., 2014) at Microsynth AG (Balgach, Switzerland) on an Illumina MiSeq platform. The reads were provided demultiplexed and trimmed. Subsequent analyses were performed using QIIME2 (Bolyen et al., 2019)^1^. Paired ends were joined with vsearch (Rognes et al., 2016) and sequences were denoised with DADA2, according to Callahan et al. (2016). Amplicon Sequence Variants (ASVs) were defined at 100% sequence identity (Callahan et al., 2017) and one representative sequence was selected for each ASV. SILVA SSU Ref database version 138 (Quast et al., 2013) was used to classify the representative sequences and obtain ASV tables at different taxonomic levels. Representative sequences for each ASV were aligned with mafft (Katoh and Standley, 2013) and phylogeny was calculated with the fasttree method (Price et al., 2010). An in-depth phylogenetic analysis was performed for reads assigned to unclassified members of the genus *Pseudomonas*, which was the most abundant in the most contaminated groundwater sample and could be putatively involved in arsenic transformations. Reads were aligned against the Genbank database with BLASTn. Reads were aligned to the closest relative sequences according to MUSCLE algorithm (Edgar, 2004) using the MEGA software version 6 (Tamura et al., 2013). The phylogenetic tree was calculated with the Maximum Likelihood method based on the Tamura-Nei model (Tamura and Nei, 1993).

### Putative Functional Profiling of Groundwater Microbial Populations

Within the ASV tables obtained from the 16S rRNA genes Illumina libraries, bacterial species putatively involved in arsenic, iron, manganese, and sulfur redox reactions were extracted and inferred, according to literature data as well as the presence of genes encoding enzymes involved in arsenic transformation in the genomes deposited in the NCBI. The metabolic pathways included in this analysis were: dissimilative reduction of As(V), Fe(III), SO_4_^2–^ and Mn(IV), chemolithoauthotrophic As(III), ferrous iron [Fe(II)], sulfur and Mn(II) oxidation, detoxification reactions of As(V) reduction, As(III) oxidation, and As(III) methylation (see Supplementary Data Sheet 1 for details).

### Real Time qPCR Amplification

Real Time quantitative PCR (qPCR), applied to environmental DNA extracted from groundwaters, was used to quantify the following phylogenetic and functional marker genes: total bacterial and archaeal 16S rRNA genes, Fe(III)-reducing families *Geobacteraceae* and *Shewanellaceae*, Fe(II)-oxidizing family *Gallionellaceae*, bi-sulfite reductase (*dsrA*) of dissimilatory sulfate reducing-bacteria, L subunit of the ribulose-1,5-bisphosphate carboxylase/oxidase (RuBisCo) (*cbbL*), arsenite oxidase (*aioA*), arsenate reductase (*arsC*), dissimilatory arsenate reductase (*arrA*), and As(III) methylase (*arsM*). The target genes evaluated with qPCR are listed in Supplementary Data Sheet 2. All reactions were performed in a final volume of 20 μL containing 10 ng of template DNA, each primer at concentrations according to Supplementary Data Sheet 2 and 1× Titan HotTaq EvaGreen^®^ qPCR Mix (Bioatlas). The reactions were performed on a 96-wells QuantStudio^TM^ 3 thermocycler (Thermo Fisher Scientific, Rockford, IL, United States), incubated under thermal conditions for each primer couple according to related references provided in Supplementary Data Sheet 2. The relative abundance of functional genes was calculated in relation to bacterial 16S rRNA gene copy number, according to López-Gutiérrez et al. (2004).

### Enrichment of Arsenic-Transforming Bacteria

The method described by Battaglia-Brunet et al. (2002) was used to enrich heterotrophic and autotrophic As(III)-oxidizing bacteria and aerobic As(V)-reducing bacteria from groundwater samples. The growth medium, hereafter referred as BBWM, included: solution A [KH_2_PO_4_ 0.04 g L^–1^; K_2_HPO_4_ 0.04 g L^–1^; NaCl 1.0 g L^–1^; (NH_4_)_2_SO_4_ 0.4 g L^–1^; pH adjusted to 6.5], solution B (CaCl_2_ 0.2 g L^–1^; MgSO_4_ 0.2 g L^–1^), vitamin solution (0.005 g L^–1^ para-aminobenzoic acid, 0.005 g L^–1^ biotin, 0.002 g L^–1^ folic acid, 0.001 g L^–1^ pyridoxine-HCl, 0.005 g L^–1^ riboflavin, 0.005 g L^–1^ thiamine, 0.005 g L^–1^ nicotinic acid, 0.005 g L^–1^ pantothenic acid, 0.0001 g L^–1^ vitamin B12), and trace element (1.5 g L^–1^ FeCl_2_⋅4H_2_O, 0.19 g L^–1^ CoCl_2_⋅6H_2_O, 0.1 g L^–1^ MnCl_2_⋅4H_2_O, 0.07 g L^–1^ ZnCl_2_, 0.062 g L^–1^ H_3_BO_3_, 0.036 g L^–1^ Na_2_MoO_2_⋅2H_2_O, 0.024 g L^–1^ NiCl_2_⋅6H_2_O, 0.017 g L^–1^ CuCl_2_⋅2H_2_O). For As(III)-oxidizing and As(V)-reducing enrichments, 2 mL of 65 g L^–1^ sodium As(III) (NaAsO_2_) and 30 mL of 156 g L^–1^ disodium As(V) (Na_2_HAsO_4_) were added, respectively. Autotrophic or heterotrophic growth was achieved by the addition of either NaHCO_3_ 80 g L^–1^ or 20% (w/v) sodium gluconate, respectively.

Anaerobic dissimilatory As(V)-reducing bacteria were enriched according to Niggemyer et al. (2001). The growth medium included: 2.5 g L^–1^ NaHCO_3_, 1.5 g L^–1^ NH_4_Cl, 0.6 g L^–1^ KH_2_PO_4_, 0.1 g L^–1^ KCl, 1 mL of trace elements solution, 5 mL of vitamin solution, 10 mL of cysteine (C_3_H_10_CNO_3_S) 15 g L^–1^, 20 mL of sodium lactate solution (C_3_H_5_NaO_3_) 50 g L^–1^, and 0.06 mL of 156 g L^–1^ disodium As(V) (Na_2_HAsO_4_). The medium was assembled anaerobically. NaHCO_3_, As(III) and As(V) solutions, sodium gluconate, cysteine, sodium lactate, and vitamin solution were sterilized by filtration over a 0.22 μm filter (Millipore, MA, United States). All other components were sterilized by autoclaving. The enrichment cultures for each metabolism were prepared in triplicate, mixing groundwater sample and culture medium in 1:1 proportion into flasks.

As(III)-methylating bacteria enrichments were set up according to Maguffin et al. (2015). Groundwater sample was added with 2.5 g L^–1^ formate, 0.46 g L^–1^ ethanol, 2.0 mg L^–1^ As(V) solution and goodies solution (0.54 g L^–1^ MgO, 0.1 g L^–1^ CaCO_3_, 0.475 g L^–1^ FeSO_4_⋅7H_2_O, 0.072 g L^–1^ ZnSO_4_⋅7H_2_O, 0.056 g L^–1^ MnSO_4_⋅4H_2_O, 0.0125 g L^–1^ CuSO_4_⋅5H_2_O, 0.014 g L^–1^ CoSO_4_⋅7H_2_O, 3 mg L^–1^ H_3_BO_3_, 3 g L^–1^ MgSO_4_, and 0.1% HCl).

All enrichment cultures were incubated under shaking (90 rpm) at 15°C, which was the average temperature of sampled groundwaters.

The ability either to reduce As(V) or to oxidize As(III) in subsequent transplants of enrichment cultures, was tested by spectrophotometric analysis according to the method of Dhar et al. (2004).

### As(III) Oxidation Kinetics

Kinetics of As(III) oxidation were analyzed in autotrophic and heterotrophic As(III)-oxidizing enrichment cultures. Culture incubation conditions were the same as for enrichment procedures. As(III) was added as 2 mL and 1 mL of 65 g L^–1^ sodium As(III) (NaAsO_2_) in autotrophic and heterotrophic conditions, respectively (section “Selection of Active Arsenic-Transforming Enrichment Cultures”). Cell suspensions were collected to follow cell growth and to determine the concentrations of inorganic [As(V) and As(III)] arsenic species.

Total arsenic and inorganic arsenic species were measured by ICP-MS according to Kim et al. (2007). Total arsenic was determined in 5 mL of samples, previously acidified with 2% HNO_3_ (v/v). As(III) and As(V) concentrations were determined in 5 mL of samples filtered with WATERS Sep-Pak^®^ Plus Acell Plus QMA cartridge (Waters, MA, United States). As(III) was collected after passing through the cartridge, whereas As(V) was retained and subsequently washed and collected with 0.16 M HNO_3_. Standards of As for concentrations ranging from 0 to 1 mg L^–1^ were prepared from sodium As(III) (NaAsO_2_; Sigma Aldrich, United States) solution.

Bacterial growth was determined by either spectrophotometric method (OD_600 nm_) or 4,6-diamine-2- phenylindole (DAPI) staining for heterotrophic and autotrophic As(III)-oxidizing enrichment cultures, respectively. For DAPI count, 5 mL of bacterial culture was vortexed in 15 mL tubes with glass beads for 5 min. The samples were centrifuged at 13,000 rpm at 4°C for 5 min and the supernatant was discarded. The bacterial pellet was resuspended in 1 mL paraformaldehyde 3% (w/v) and incubated at 4°C for 3 h. The samples were then washed 3 times with 1× PBS for 3 times and resuspended in saline solution (NaCl 9 g L^–1^). DAPI solution was added to the samples to a final concentration of 5 μg L^–1^, and incubated in the dark for 15 min at room temperature. The samples were immobilized on black 0.2 mm Isopore^TM^ GTBP membrane filters (Millipore), and mounted on glass slides with Citifluor antifadent oil (Citifluor, Electron Microscopy Sciences, Hatfield, PA, United States). Microbial cells were observed on a fluorescence microscope (Zeiss Axioskop, Germany), supplied with a Mercury Short Arc HBO 50W/ACL2 OSRAM UV lamp and Zeiss 1 filter set, and counted on 100 microscopic fields using a calibrated grid.

Specific As(III) oxidation rates (i.e., μmol of As(V) produced in 1 day by each cell) in autotrophic conditions, were calculated according to the following formula:

$$\begin{array}{c} SpecificAs\left(III\right)oxidationrate=\\ \frac{\begin{array}{c} ReleasedAs\left(V\right)\left(\mu molmL^{-1}\right)\cdot Cellincreaseatexponential\\ phase\;\left(cellsmL^{-1}\right) \end{array}}{Incubationtime\left(days\right)} \end{array}$$

Non-inoculated flasks were prepared to follow the abiotic transformation of arsenic. Each condition was tested in triplicate.

### Statistical Analysis

The correlations within physicochemical parameters and between As(III) oxidation rates and physicochemical parameters were tested by calculating Pearson correlation coefficient at *p* < 0.05, using the base package of the R program, v.3.6.0 (R Core Team, 2015), after log transformation of data. The statistical analyses of Illumina 16S genes library data were performed with QIIME2 and with the R package vegan version 2.5–5 (Oksanen et al., 2018). Alpha diversity was calculated for the samples upon rarefaction of the datasets. Species richness was estimated calculating the Shannon-Weaver index (Shannon and Weaver, 1949), whereas species evenness was calculated according to Pielou’s algorithm (Pielou, 1966). Non-Metric Multidimensional Scaling (NMDS) was performed on Bray-Curtis dissimilarities calculated from rank-transformed abundance data (Conover and Iman, 1981; Faith et al., 1987). Physicochemical data were fitted to the NMDS plot after log transformation. Data produced by qPCR were tested for statistical significance with two-way ANOVA, Tukey’s b, Duncan, and *t*-test at *p* < 0.05, using the base package of the R program.

## Results

### Physicochemical Characterization of Groundwater Samples

The results of the physicochemical characterization of groundwater samples are listed in Table 2 and Supplementary Table 1. The groundwaters analyzed in this study were indicative of reducing and oligotrophic environments according to oxidation-reduction potential, dissolved oxygen concentration, and organic carbon concentration. Total arsenic concentration in the groundwaters ranged between 28.9 and 193.7 μg L^–1^, above drinking water limit of 10 μg L^–1^ (Table 2). As(III) was the predominant inorganic species in the most contaminated samples BS and CR2, whereas As(V) prevailed in less contaminated samples. Methylated arsenic forms were not detected. In samples LO, CR1, CR2, and BS, iron and manganese exceeded the parameters indicated by the European Union of 0.2 mg L^–1^ and 0.05 mg L^–1^, respectively (World Health Organization [WHO], 2011; European Commission Directive, 2015). Fe(II) was the predominant iron species in all samples. SO_4_^2–^ was generally higher in less contaminated samples. Sulfides were never retrieved. In terms of nitrogen dynamics, the samples could be gathered into two groups: high NH_4_^+^ (CR2, MN, BS) and low NH_4_^+^ (VA, CR1, LO, Table 2) content (World Health Organization [WHO], 1996). Nitrates were below the EU Nitrate Directive limit of 50 mg L^–1^ (European Commission Directive, 1991). Significant negative Pearson correlations were observed between pH and total iron and between Fe(II) and NO_3_^–^ (*p* < 0.05, Supplementary Table 2). On the other hand, Fe(II) and manganese and depth and As(V) were positively correlated (*p* < 0.05, Supplementary Table 2). No significant correlation was observed between depth, Eh and dissolved oxygen and between arsenic and iron (*p* < 0.05, Supplementary Table 2).

**TABLE 2:** Physicochemical characterization of groundwaters samples.

		Groundwater samples					
Parameter	Unit	LO	CR1	MN	VA	BS	CR2
T	°C	18.3	15.3	16.8	13.1	18	14.7
pH		7.66	7.61	7.88	8.14	7.43	7.62
Eh	mV	−31	−117	−140	352	−111	−94
Dissolved O_2_	mg L^–1^	6.52	3.75	4.05	12.5	5.22	0.44
Organic carbon	mg L^–1^	<5	<5	<5	<5	<5	<5
NO_3_^–^	mg L^–1^	0.6	0.3	<0.3	<0.3	2.3	<0.3
NH_4_	mg L^–1^	0.3	<0.2	1.2	<0.2	2.2	2.2
Tot As	μg L^–1^	28.9 ± 0.36	33.6 ± 0.27	34.8 ± 0.33	47.4 ± 0.26	97.9 ± 3.50	193.7 ± 3.58
As(III)	μg L^–1^	0.4 ± 0.0	8.3 ± 0.4	1.4 ± 0.1	10.3 ± 1.8	68.5 ± 0.8	131.3 ± 1.8
As(V)	μg L^–1^	23.1 ± 0.9	13.3 ± 0.7	33.7 ± 2.4	29.2 ± 1.5	21.0 ± 0.9	49.7 ± 0.7
Tot Fe	mg L^–1^	0.68 ± 0.12	0.93 ± 0.01	0.25 ± 0.06	0.16 ± 0.07	1.47 ± 0.08	0.73 ± 0.08
Fe(II)	mg L^–1^	0.46 ± 0.04	0.81 ± 0.02	0.14 ± 0.01	0.19 ± 0.07	1.34 ± 0.02	0.59 ± 0.00
Mn	μg L^–1^	77.10 ± 0.85	140.53 ± 1.97	0.75 ± 0.05	22.62 ± 1.36	83.12 ± 1.83	50.63 ± 0.65
SO_4_^2–^	mg L^–1^	7.0 ± 0.0	30.50 ± 2.12	2.7 ± 0.28	17.0 ± 0.0	0.58 ± 0.46	0.25 ± 0.0

### Prokaryotic Abundance Analyzed by Fluorescent Cell Staining

**FIGURE 1:**
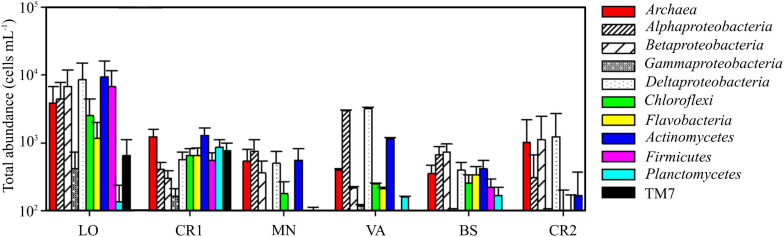
Total abundance of different microbial populations in the groundwater samples, quantified by CAtalyzed Reporter Deposition Fluorescence *in situ* Hybridization (CARD-FISH).

Sample CR2, with the highest arsenic content, showed the lowest concentration of prokaryotic cells, accounting for 0.7 ⋅ 10^4^ cells mL^–1^ (Table 3 and Supplementary Figure 2). On the other hand, sample LO, with the lowest amount of arsenic, was the richest sample, with 8.7 ⋅ 10^4^ cells mL^–1^. In this sample, 87.7% of the stained cells were classified as HNA, whereas in the other samples LNA and HNA accounted on average for 50% of the total. The microbial community in LO was dominated by *Actinomycetes*, *Deltaproteobacteria*, *Firmicutes*, *Betaproteobacteria*, *Alphaproteobacteria*, and *Archaea*, whereas in CR2 the community was more homogeneous, with only *Deltaproteobacteria*, *Betaproteobacteria*, and *Archaea* (Figure 1). Differently from CR2 and despite high arsenic concentration, in BS the microbial community was more heterogeneous.

**TABLE 3:** Prokaryotic abundance and HNA and LNA cell fractions as measured by flow cytometry in the groundwater samples.

Sample	Prokaryotic abundance (10^4^ cells mL^–^^1^)	LNA cells (%)	HNA cells (%)
LO	8.7	12.2	87.7
CR1	1.7	53.0	46.9
MN	0.8	57.4	42.6
VA	1.5	50.5	49.5
BS	1.2	54.4	45.6
CR2	0.7	48.4	51.6

### Microbial Communities and Putative Functional Profiling in Groundwater Samples

**FIGURE 2:**
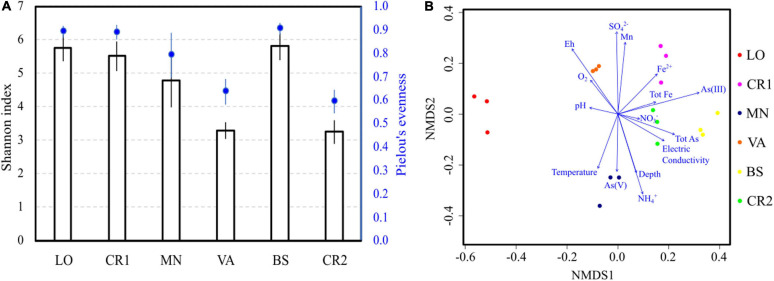
Alpha **(A)** and beta **(B)** diversity in the groundwater samples. Species richness was evaluated calculating the Shannon-Weaver index (Shannon and Weaver, 1949), whereas species evenness was estimated according to Pielou’s algorithm (Pielou, 1966) **(A)**. Beta diversity was evaluated by Non-metric MultiDimensional Scaling (NMDS); log-transformed chemical data were fitted to the ordination **(B)**.

Illumina sequencing of 16S rRNA genes in the analyzed groundwaters produced on average 9,500 reads from each sample, with 96 ± 16 identical ASVs (Supplementary Table 3). Species richness and evenness followed trends similar to those observed in active microbial population analysis, with samples VA and CR2 having the lowest number of species, unevenly distributed (Figure 2A). Despite high arsenic concentration, sample BS showed high richness and evenness values, similarly to what observed with CARD-FISH. In terms of composition, NMDS revealed that sample LO was the most divergent (Figure 2B), although this divergence was not clearly explained by any physicochemical parameter. The microbial composition of the most arsenic-contaminated samples CR2 and BS and of sample CR1 were driven by arsenic, iron and manganese concentrations. In sample VA, the highest Eh and DO levels significantly explained the retrieved microbial diversity. The concentration of NH_4_^+^ and the Eh value (Table 2) were opposite drivers, with high NH_4_^+^ in low-Eh samples (CR2, BS, MN). These parameters were responsible for a strong differentiation in the microbial communities (Figure 2B).

**FIGURE 3:**
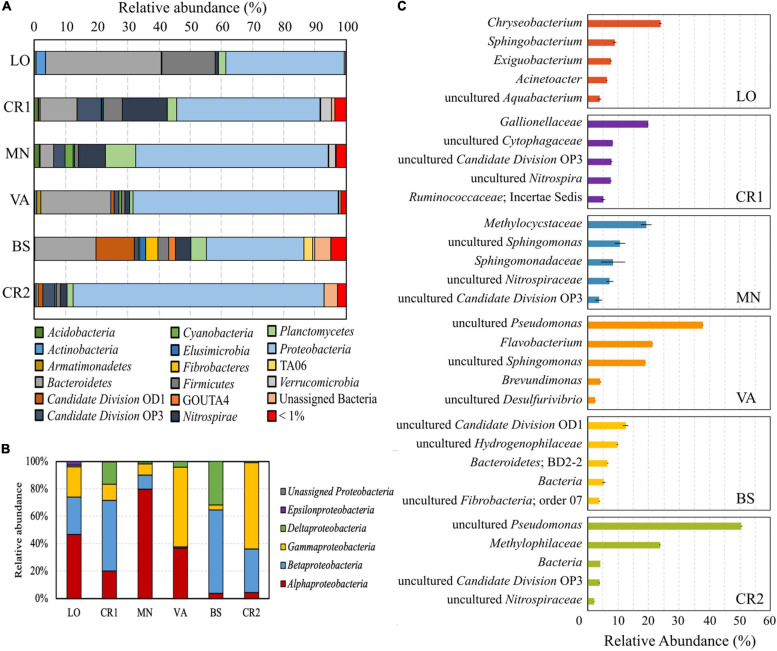
Composition of the microbial communities retrieved in the groundwater samples. Colored pile bars show the percentage of microorganisms at the phylum level **(A)** and relative abundance of reads classified within the class Proteobacteria **(B)**; colored bars **(C)** indicate the relative abundance of the 5 most abundant taxa at the genus level for each sample.

In the analyzed groundwater samples, 27.09–72.2% of the reads were unassigned at different taxonomic levels. Whereas VA, MN, and LO had the highest number of unclassified genera, in sample CR2 72.91% of the reads were assigned to classified genera (Supplementary Figure 3). All samples were dominated by the phylum *Proteobacteria* (Figure 3A), with *Alpha*-, *Beta-*, and *Gammaproteobacteria* making up more than 65% of the total phylum (Figure 3B). *Deltaproteobacteria* accounted for 31.67 and 16.39% of total *Proteobacteria* in samples BS and CR1, respectively, both having the highest concentration of Fe(II) (Table 2). In samples VA, LO, CR1, and BS, the second most abundant phylum was *Bacteroidetes*, followed by *Firmicutes*, *Nitrospirae*, *Candidate Division* OD1, *Candidate Division* OP3, and *Planctomycetes*.

In terms of species abundance, samples CR2 and VA showed few dominant taxa, whereas species in samples LO, CR1, BS, and MN were more evenly distributed (Figure 3C), in accordance with their higher evenness (Figure 2A). In the most contaminated sample CR2, unclassified species within the genus *Pseudomonas* accounted for 52% of the total reads, followed by unclassified members of the methylotrophic family *Methylophilaceae*. Unclassified *Pseudomonas* were also dominant in sample VA, followed by *Flavobacterium* and unclassified *Sphingomonas*. In samples LO, CR1, BS, and MN *Chryseobacterium* (25%), *Gallionellaceae* (20%), unclassified species belonging to *Candidate Division* OD1 (12%), and *Methylocystaceae* (20%) were, respectively dominant, respectively.

**FIGURE 4:**
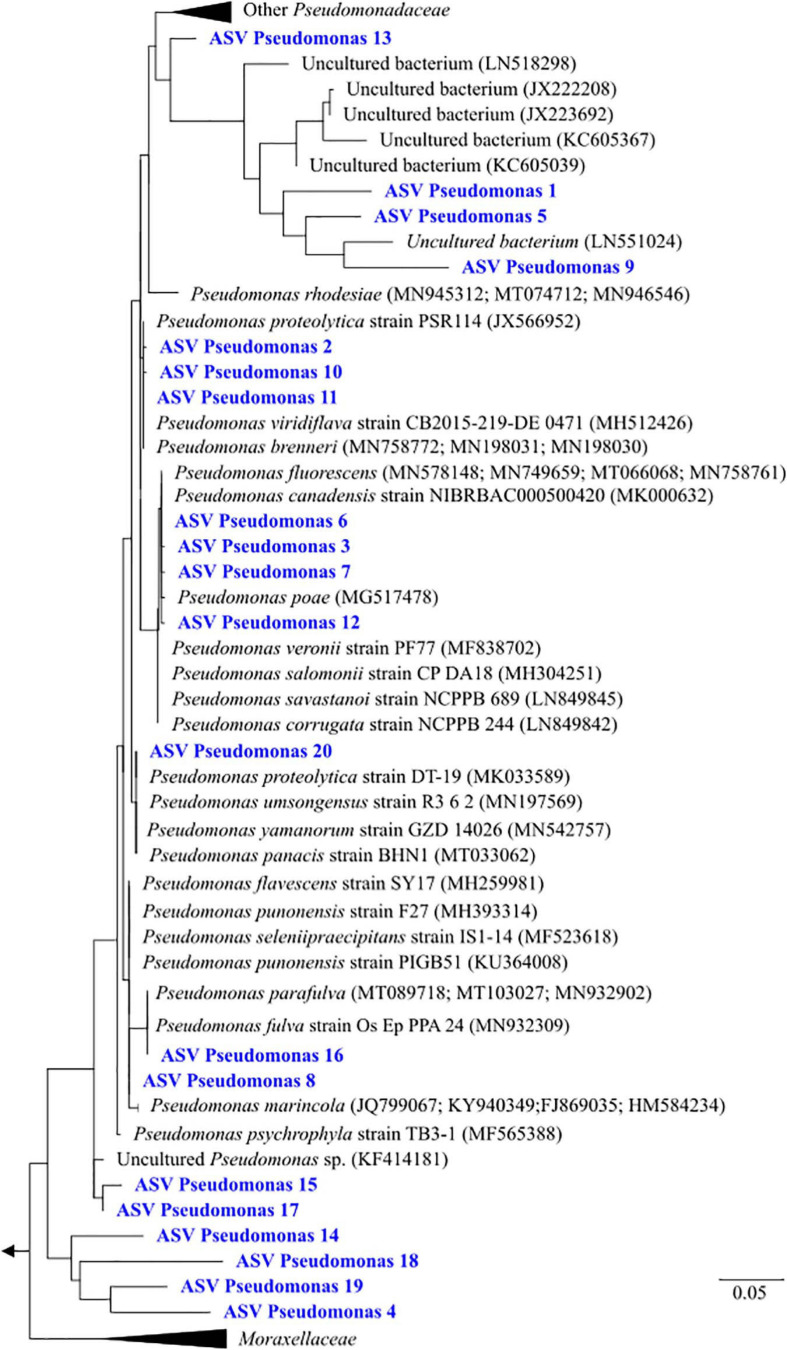
Phylogenetic analysis of Amplicon Sequence Variants (ASVs) assigned to the genus *Pseudomonas*.

Since ASVs assigned as “uncultured *Pseudomonas*” were dominant in the most contaminated sample CR2, an in-depth phylogenetic analysis was performed for this taxon. In total, 20 ASVs were assigned as “uncultured *Pseudomonas*.” Among these, 10 ASVs clustered to different characterized species of cultured strains, although phylogeny based only on 16S rRNA gene was not sufficient to determine the exact species classification (Figure 4). The other 10 ASVs (ASVs *Pseudomonas* 1, 4, 5, 9, 13, 14, 15, 17, 18, 19) had a low identity (90–96%) with characterized species. Instead, they clustered with uncultured closest relatives evidenced in different types of contaminated and non-contaminated groundwaters (Flynn et al., 2013; Alessi et al., 2014; Gülay et al., 2014).

According to putative functional profiling, in samples VA and CR2, the highest abundance of As(V)-reducing for detoxification purpose, As(III)-oxidizing, As(III)-methylating as well as Fe(II)-oxidizing, Mn(IV)-reducing and Mn(II)-oxidizing genera was retrieved (Supplementary Figure 4). In LO and in MN [with lower arsenic concentration, but higher As(V) proportion], a higher abundance of SOB was retrieved compared to the other samples (Supplementary Figure 4). In sample BS, low relative abundances were reported for all functional groups, with only dissimilatory sulfate-reducing bacteria reaching 2% of the total community.

### Quantification of Taxonomic and Functional Genes

**FIGURE 5:**
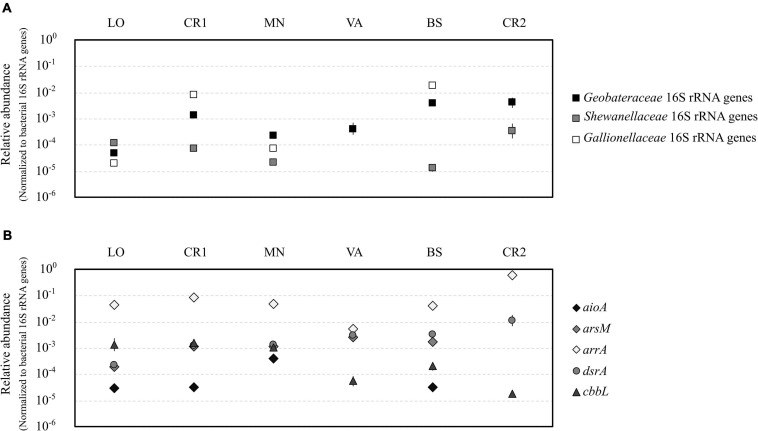
Relative abundance of iron-cycling bacteria belonging to the families *Geobacteraceae*, *Shewanellaceae*, and *Gallionellaceae* **(A)** and of genes encoding the A subunit of dissimilatory bi-sulfite reductase (*dsrA*), arsenite oxidase (*aioA*), arsenite methylase (*arsM*), dissimilatory arsenate reductase (*arrA*), and the A subunit of bisulfite reductase and ribulose-1,5-bisphosphate carboxylase/oxidase (*cbbL*) **(B)**. All abundances were normalized to the abundance of bacterial 16S rRNA genes. Statistical significant groups based on ANOVA (Tukey’s test, *p* ≤ 0.05) were reported in Supplementary Table 5.

Quantification of gene copies for bacterial and archaeal 16S rRNA genes confirmed the highest microbial load in samples LO and CR1 (10^7^ gene copies L^–1^, Supplementary Figures 5A,B), whereas sample CR2 showed the lowest microbial load (10^4^ gene copies L^–1^). On the contrary, samples LO and CR1 were the richest in terms of bacterial and archaeal 16S rRNA genes, respectively. In terms of relative abundance, the Fe(III)-reducing families *Geobacteraceae* and *Shewanellaceae* were significantly more abundant in samples with the highest As concentration (BS and CR2) (Figure 5A). On the other hand, the Fe(II)-oxidizing family *Gallionellaceae* was significantly more abundant in samples with the highest Fe(II) concentration (CR1 and BS) (Figure 5A). *aioA* genes for arsenite oxidase were significantly more abundant in sample MN, whereas in VA and CR2 they were not amplified (Figure 5B). With the only exception of sample CR2, *arsM* genes for arsenite methyltransferase were present in all samples, although methylated arsenic species were never retrieved (Figure 5B). *arrA* genes for arsenate respiratory reductase were the most abundant among the analyzed functional genes, with significantly higher abundance in CR2 (Figure 5B and Supplementary Table 4). On the contrary, none of the tested primer couples amplified arsenate reductase *arsC* in any of the analyzed groundwater samples. In sample CR2, *dsrA* genes for dissimilatory bi-sulfate reductase were significantly more abundant with respect to all other samples (Figure 5B). The presence of autotrophic microorganisms was evidenced by the amplification of *cbbL* gene for RuBisCo in all samples, being most abundant in the least contaminated samples (LO, CR1, and MN).

### Arsenic-Transforming Bacteria Enriched From Groundwater Samples

In order to demonstrate the presence of microbial populations that are potentially active in biogeochemical cycle of the metalloid, selective enrichment cultures were established from groundwater samples (Table 4). In anaerobic conditions, As(V) dissimilative reduction was achieved in two 60 day long subsequent transplants of MN, BS, and CR2 groundwaters. Successive transplants failed to grow, although *arrA* genes for arsenate respiratory reductase were present in the environmental DNAs (Figure 5). In aerobic conditions, heterotrophic As(V) reduction for detoxification purpose was selected from VA and CR2 groundwaters. Aerobic As(III)-oxidizing populations were found in all samples, being chemolithoautotrophic metabolism selected from all groundwaters, whereas heterotrophic metabolism selected only from the most contaminated samples BS and CR2. Anaerobic As(III) oxidation coupled with NO_3_^–^ reduction was reached in 90 days in one sample. As(III)-methylating cultures were not displayed at the studied sites.

**TABLE 4:** Arsenic metabolisms selected in groundwater microbial communities: arsenic transformation analyzed spectrophotometrically and microbial growth measured at optical density OD_600 *nm*_.

Sample	As(V)-respiring bacteria	Aerobic As(V)-resistant bacteria	Nitrate-reducing As(III) oxidizers	Heterotrophic As(III) oxidizers	Autotrophic As(III) oxidizers
LO	−	−	−	−	+
CR1	−	−	+	−	+
MN	+	−	−	−	+
VA	−	+	−	−	+
BS	+	−	−	+	+
CR2	+	+	−	+	+

*−, negative growth and absence of arsenic transformation; +, positive growth and presence of arsenic transformation.*

**FIGURE 6:**
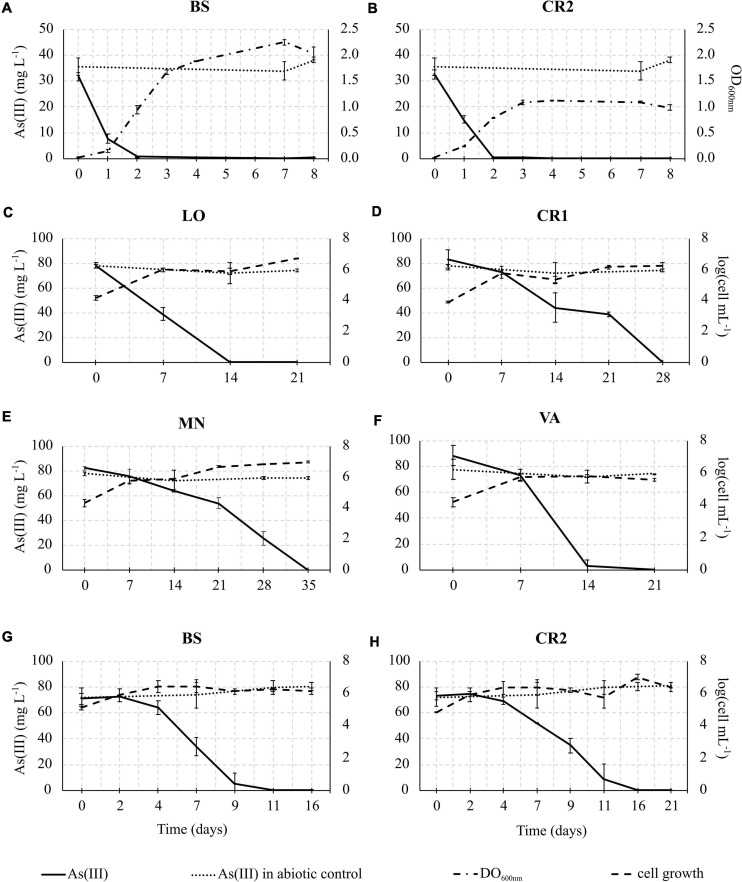
As(III) oxidation kinetics in heterotrophic enrichment cultures set up from BS **(A)** and CR2 **(B)** samples; As(III) oxidation kinetics in autotrophic enrichment cultures set up from LO **(C)**, CR1 **(D)**, MN **(E)**, VA **(F)**, BS **(G),** and CR2 **(H)** groundwater samples.

In consideration of the relevance of As(III) oxidation processes exploitable in biological water treatment plants, As(III) oxidation kinetics study was conducted on the selected enrichment cultures (Figure 6). In heterotrophic conditions, oxidation of 30 mg L^–1^ As(III) to As(V) was completed within 48 h of incubation in BS and CR2 (Figures 6A,B). In chemolithoautotrophic conditions, oxidation of 80 mg L^–1^ As(III) to As(V) was completed at different incubation periods: 9–11 days for BS and CR2 and more than 11 days for LO, VA, CR1 and MN (Figures 6C–H). In parallel to metalloid oxidation, the bacterial cell number increased.

**FIGURE 7:**
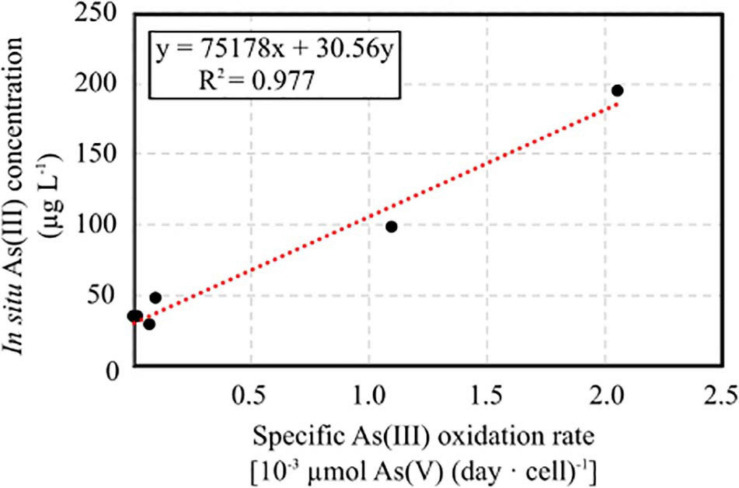
Linear correlation between specific As(III) oxidation rate calculated on enrichment cultures set up from groundwater samples and As(III) concentration measured *in situ*.

The specific As(III) oxidation rate was significantly higher in BS and CR2 with respect to the other samples (Table 5). Linear correlation (*p* < 0.05, Figure 7) between specific oxidation rate and As(III) concentration measured at the parental sites, indicated that local As(III) concentration was a relevant factor for selecting specific bacterial populations.

**TABLE 5:** Arsenite oxidation rates in autotrophic As(III)-oxidizing enrichment cultures.

Sample	Exponential phase endpoint (days)	Cells mL^–^^1^ at endpoint	Daily growth rate (%)	Specific As(III) oxidation rate [mmol As(V) (day ⋅ cells) ^–^^1^]
LO	14	7.87 ⋅ 10^5^	4.30	7.26 ⋅ 10^–5^
CR1	28	1.72 ⋅ 10^6^	7.98	2.41 ⋅ 10^–5^
MN	35	9.31 ⋅ 10^6^	14	3.01 ⋅ 10^–6^
VA	14	6.62 ⋅ 10^5^	3.33	9.84 ⋅ 10^–5^
BS	9	1.72 ⋅ 10^6^	1.24	1.11 ⋅ 10^–3^
CR2	11	1.07 ⋅ 10^7^	9.79	2.06 ⋅ 10^–3^

## Discussion

**FIGURE 8:**
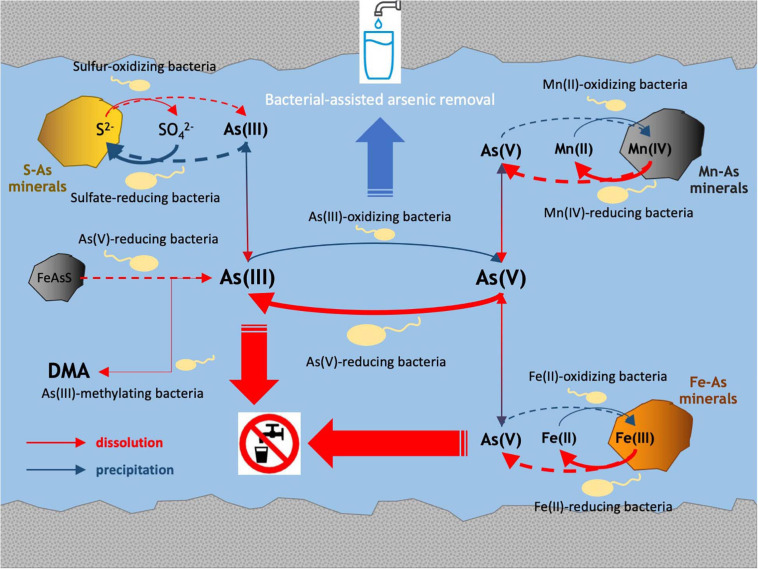
Main processes involved in arsenic biogeochemistry in Po Plain contaminated aquifers.

The multidisciplinary approach implemented in the present study revealed relationships between *in situ* microbial communities, arsenic-cycling bacteria and physicochemical characteristics in the Po Plain metalloid-contaminated aquifer systems. The main processes involved in arsenic biogeochemistry retrieved in these ecosystems are drawn in Figure 8.

Arsenic concentration and speciation were the main drivers of abundance and composition of microbial communities inhabiting the analyzed groundwaters. In LO, the shallowest sample, with the lowest arsenic concentration and with different usage with respect to all other samples (livestock), microbial populations were the most abundant, diverse, and active, as confirmed by fluorescent cell staining and NMDS analysis. On the other hand, the most contaminated sample CR2 showed the lowest diversity and richness. Furthermore, in sample CR2 more than 75% of the retrieved taxa were related to cultivated species, whereas in the other samples this fraction accounted for less than 30% of the total. A high number of unclassified species was previously revealed by Illumina 16S rRNA gene sequencing performed on Po Plain groundwaters (Cavalca et al., 2019) as well as in Central Italy (Crognale et al., 2017). These outcomes confirm that Italian groundwaters represent a source for novel microbial species, with great potential for bioremediation purposes in arsenic-rich environments.

Although groundwaters had low Eh, they were not anoxic, since O_2_ ranged between 0.44 and 12.5 mg L^–1^. In fact, a number of bacterial taxa known to be aerophilic or microaerophilic, like *Gallionellaceae* were identified. This aspect suggests that Po Plain deep aquifers are not confined systems and element exchange might occur to a certain extent, as observed in other European aquifers (Bochet et al., 2020; Voisin et al., 2020).

Samples CR2 and VA were dominated by classified and unclassified members of the genus *Pseudomonas* (Figure 3C). The dominance of this genus was found in highly arsenic-contaminated aquifers in Hetao basin (Li et al., 2015). This genus is among the widest bacterial genera and includes several species with versatile metabolic properties (Palleroni, 2015). *Pseudomonas* spp. are common inhabitants of groundwaters all over the world (Guo et al., 2019), and have been exploited in several bioremediation experiments for their wide metabolic capacities (Bahar et al., 2016; Khodaei et al., 2017; Ko and Kong, 2017; Poi et al., 2018). A specific cluster of *Pseudomonas* ASVs retrieved in the present study was closely related to planktonic inhabitants of pristine and uranium-contaminated environments (Flynn et al., 2013; Alessi et al., 2014). Different *Pseudomonas* species have been demonstrated to perform reduction, oxidation and methylation of arsenic (Freikowski et al., 2010; Koechler et al., 2015; Zhang et al., 2015). Indirect dissolution of arsenic was shown for a Mn(IV)-reducing *P. fluorescence* (Horvath et al., 2014). The dominance of these microorganisms in the most contaminated sample (CR2) strongly suggest a possible role in direct and/or indirect dissolution of arsenic, although the ability to perform As(III) oxidation cannot be excluded. Further studies are needed to characterize these *Pseudomonas* species, in order to clarify the ecological role and potential exploitation of these uncharacterized species for arsenic bioremediation.

In all the aquifers characterized in this study, reducing conditions were verified. Metal dissolution was confirmed by the presence of reduced species like NH_4_^+^, Fe(II), As(III), and manganese, although a significant correlation between Eh and metal concentrations could not be evidenced. Nonetheless, these parameters are indicative of reductive dissolution of arsenic-bearing phases common in Po Plain groundwater sediments, such as iron oxy-hydroxides (goethite, magnetite, ferrihydrite, lepidocrocite), and MnO_2_, driven by the degradation of peat layers in the alluvial sediments (Rotiroti et al., 2021). In samples with higher arsenic concentrations (CR2 and BS), functionality inferred by Illumina 16S rRNA genes sequencing indicated that bacterial genera related to arsenic-cycling [As(V) reduction, As(III) oxidation and methylation] as well as manganese reduction and iron oxidation, were abundant. The presence of *arrA* genes and of Fe(III)-reducing microorganisms belonging to *Geobacteraceae* and *Shewanellaceae* was also revealed by qPCR. Furthermore, dissimilatory As(V)-reducing bacteria were enriched from the most contaminated samples, where As(III) was the predominant species. These microorganisms are widely known for their promotion of arsenic solubilization from iron minerals (Ohtsuka et al., 2013; Danczak et al., 2019). These data confirm the hypothesis that in the analyzed groundwaters microbial populations can directly promote the dissolution of arsenic.

Samples CR2, BS, and MN were characterized by high NH_4_^+^ concentrations. These conditions are typically found at low Eh, due to the activity of anammox and denitrifiers producing NH_4_^+^ (Paredes et al., 2007). In the same samples, also Fe(III)-reducing bacteria were abundant. NH_4_^+^ oxidation coupled with Fe(III) reduction (i.e., Feammox; Yang et al., 2012) was proposed to be one of the main driving force for arsenic dissolution in groundwater aquifer of Hetao Basin, China (Xiu et al., 2020). In accordance with the present data, NH_4_^+^ oxidation coupled with to Fe(III) reduction could explain part of arsenic dissolution also in the Po Plain. Further studies are needed to elucidate the occurrence and extent of these processes in Italian aquifers.

Members of the phylum *Nitrospirae* and *Candidate Division* OD1 were abundant in CR1 and BS Illumina 16S libraries, respectively. *Nitrospirae* includes a variety of still poorly characterized microorganisms, known to be involved in nitrogen, iron and sulfur cycle and to live in chemolithoautotrophic conditions (Daims, 2014). Recently, several putative uncultured *Nitrospirae* species were revealed by shotgun metagenome sequencing in other subsurface environments (Danczak et al., 2019). Interestingly, in the metagenome-assembled genome of some of these putative novel species, *arrA* genes for As(V) respiration were present, suggesting a possible role in arsenic dissolution in groundwater. These microorganisms might play a crucial role in nitrogen cycling as well as arsenic dissolution in the analyzed aquifers. *Candidate Division* OD1 were found in suboxic pond (Briée et al., 2007), boreal lakes (Peura et al., 2012), groundwaters (Luef et al., 2015) as well as iron and sulfur-rich environments, where they could be involved in dissimilatory Fe(III) reduction (Borrel et al., 2010). Therefore, their presence might be related to the high concentration of iron in sample BS. The role of these phyla in arsenic dissolution should be clarified with further culturomics studies.

As suggested by Rotiroti et al. (2015, 2021), SO_4_^2–^ reduction in deep Po Plain aquifers might be involved in the formation of FeS and AsS. In fact, in the presence of soluble Fe(II) and As(III), bacterial dissimilatory reduction of SO_4_^2–^ leads to the co-precipitation of Fe(II) and sulfides to FeS and AsS minerals (Neal et al., 2001; Stanley and Southam, 2018). In the present study, the retrieval of dissimilatory sulfate-reducing bacteria, suggested by the detection in all samples of *dsrA* genes and by Illumina inferred functionality, might confirm arsenic release and attenuation dynamics in Po Plain aquifers, as modeled by Rotiroti et al. (2021).

The microbial dissolution of arsenopyrite was mainly studied in oxidizing acidic environments such as acid mine drainage. In these environments, acidic pH in combination with the oxidation performed by acidophilic Fe(II)- and S-oxidizing bacteria leads to the dissolution of arsenopyrite (process fully reviewed by Corkhill and Vaughan, 2009). However, circum-neutral pH and reducing conditions evidenced in the present study site likely exclude, or at least limit, the occurrence of such mechanisms. Indeed, the presence of little amounts of dissolved O_2_ and of Fe(II)-oxidizing and S-oxidizing bacteria suggested by both Illumina and qPCR analyses cannot exclude the possibility that such processes might occur locally within defined micro-niches. Recently, the activity of dissimilatory As(V)-reducing bacteria was shown to lead to the reductive dissolution of arsenopyrite (Kawa et al., 2019). Furthermore, Suess and Planer-Friedrich (2012) showed that at circum-neutral pH, under microoxic and reducing conditions, arsenopyrite dissolution is promoted by the presence of sulfides. In the study sites, the presence of dissimilatory As(V)-reducing bacteria might be involved in the reductive dissolution of arsenopyrite. To a lesser extent, dissimilatory SO_4_^2^–reducing bacteria could contribute to arsenopyrite dissolution by the local production of sulfide, which however are not retrieved globally in the analyzed groundwater samples.

Arsenic resistance by means of As(V) reduction encoded by *arsC* is considered widespread in arsenic-contaminated environments (Kaur et al., 2011). In this study, *arsC* could not be retrieved in any sample by qPCR, although their presence was inferred in more than 50% of the AVS obtained in three groundwater samples. This result might be imputable to mismatches between the primer couple and the microbial species living in the analyzed groundwater samples. However, in a previous study, Cavalca et al. (2019) showed that in contaminated aquifers in the province of Cremona the abundance of *arsC* ranged from 0 to 100 copies per L of groundwater, confirming that in these aquifers this target is not abundant. Besides these results, aerobic As(V) resistant bacteria were enriched in samples VA and CR2, confirming that the ARS detoxification system is present in these environments.

NMDS analysis revealed that arsenic was the main driver of microbial composition in the most contaminated samples BS and CR2. In line with this observation, heterotrophic As(III)-oxidizing and As(V)-reducing consortia were obtained only from these samples. Low availability of organic C measured in the groundwater samples suggests that chemolithoautotrophic metabolisms such as As(III) oxidation might be favored, as observed in shallow aquifers (Jewell et al., 2016). In fact, chemolithoautotrophic As(III)-oxidizing microorganisms were selected from all groundwaters, in accordance with the retrieval of *aioA* and *cbbL* genes by qPCR. A significant linear correlation between As(III) oxidation rate and *in situ* As(III) was evidenced. With respect to previous studies, As(III) oxidation from samples BS and CR2 was particularly efficient (i.e., less than 10 days) with respect to other studies (Ito et al., 2012; Fazi et al., 2016). As a confirmation, functionality inferred from 16S libraries evidenced a high potential of As(III) oxidation within bacterial species retrieved in CR2, including *Pseudomonas* spp. Overall, these data suggest that the composition of the microbial communities inhabiting the analyzed groundwaters was driven by the presence of As(III), with the selection of As(III)-oxidizing populations. Considering the relevance of As(III) oxidation processes in view of their exploitation in the bioremediation of arsenic polluted groundwaters by As(V) adsorption, large genomic and metabolic surveys performed on unexplored contaminated aquifers would be beneficial for the recovery of microbial populations that can provide essential ecosystem services.

In conclusion, microbial community structures in the studied groundwaters showed highly different patterns, likely depending on the fact that they are primed by different physicochemical properties at a local scale. The selective cultivation approach combined with genomic data showed that different metabolic properties of indigenous microbial populations have an important role on *in situ* arsenic dissolution. In Northern Italy deep aquifers, as a consequence of bacterially mediated As(III) dissolution, the selection of chemolithoautotrophic As(III)-oxidizing populations was promoted. The presence of a high abundance of previously uncharacterized species opens to possible actions for the recruitment of novel autotrophic As(III)-oxidizing microorganisms, in view of possible exploitation for bioremediation activities.

## Data Availability Statement

All sequences obtained in this study were deposited in GenBank within the PRJNA667833 Bioproject (https://www.ncbi.nlm.nih.gov/sra/PRJNA667833) and in the Dataverse repository (https://dataverse.unimi.it/dataverse/BATA).

## Author Contributions

PZ, GS, and BC performed the chemical analyses. SC, SF, and SR carried out the microscopy analyses. SA executed flow cytometry experiments. SZ conducted the molecular and statistical analyses, and wrote the original draft. MC contributed to the molecular analyses. LC and RZ performed the cultivation experiments. LC conceptualized and coordinated this study. All authors contributed to revision and editing of the submitted manuscript.

## Conflict of Interest

The authors declare that the research was conducted in the absence of any commercial or financial relationships that could be construed as a potential conflict of interest.

## Footnotes

1 https://qiime2.org/
