# Supplementary material for: Adaptation of Microbial Communities to Environmental Arsenic and Selection of Arsenite-Oxidizing Bacteria From Contaminated Groundwaters
Source: Front Microbiol. 2021 Mar 19;12:634025. doi: 10.3389/fmicb.2021.634025 (PMC8017173; doi:10.3389/fmicb.2021.634025)
Supplement: Supplementary file 2 [file Data_Sheet_2.docx]

Supplementary Material

# Supplementary Figures and Tables

## Supplementary Figures


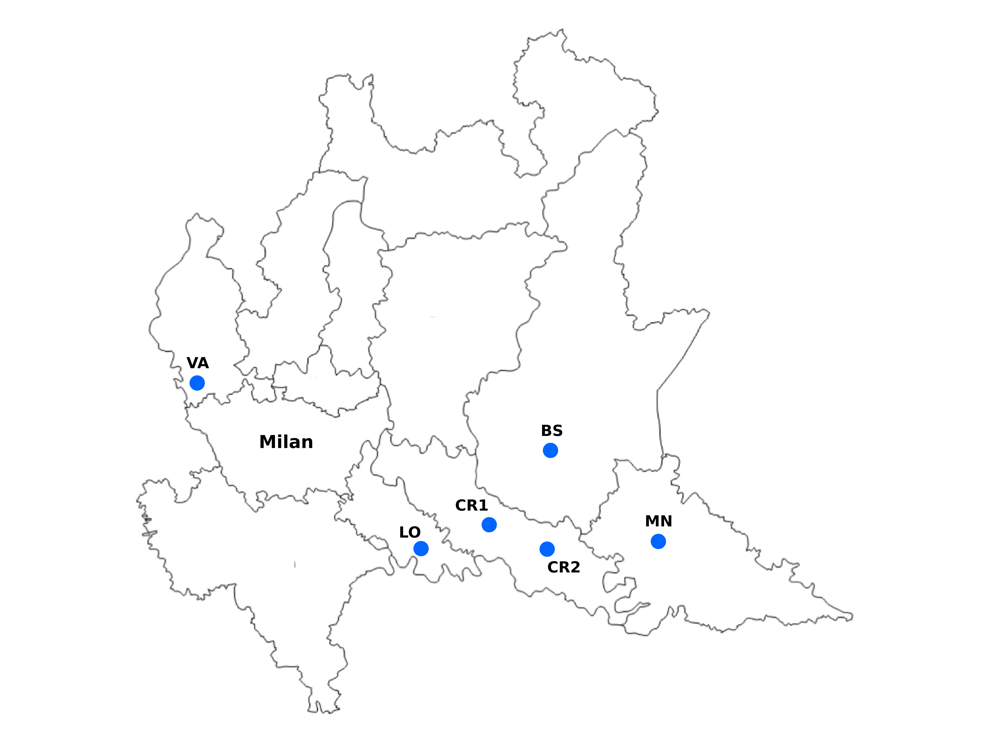


**Supplementary Figure 1.** Location of the 6 sites over Lombardy region, Northern Italy.


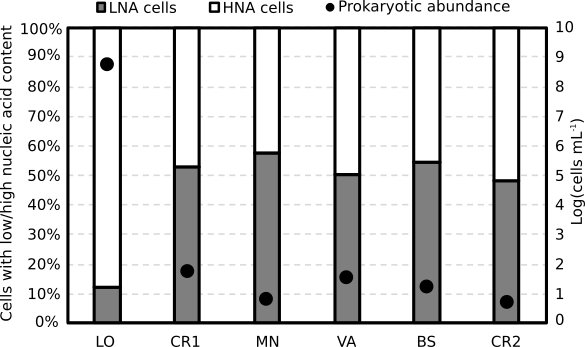


**Supplementary Figure 2.** Abundance of prokaryotic cells measured by flow cytometry, with specification of low nucleic acid (LNA) and high nucleic acid (HNA)-containing cells fractions.


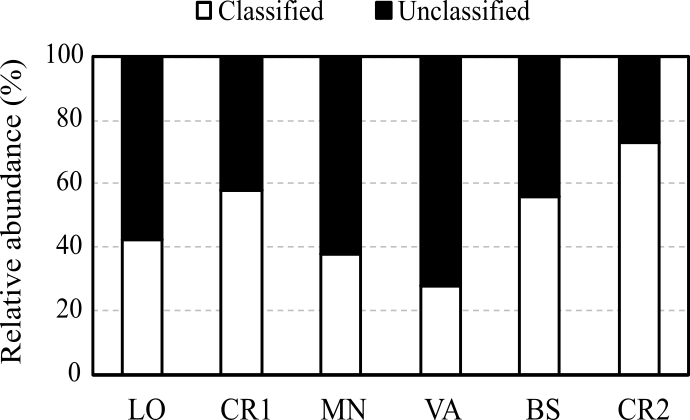


**Supplementary Figure S3.** Relative abundance of classified (white bars) vs unclassified (black bars) bacterial taxa at genus level.


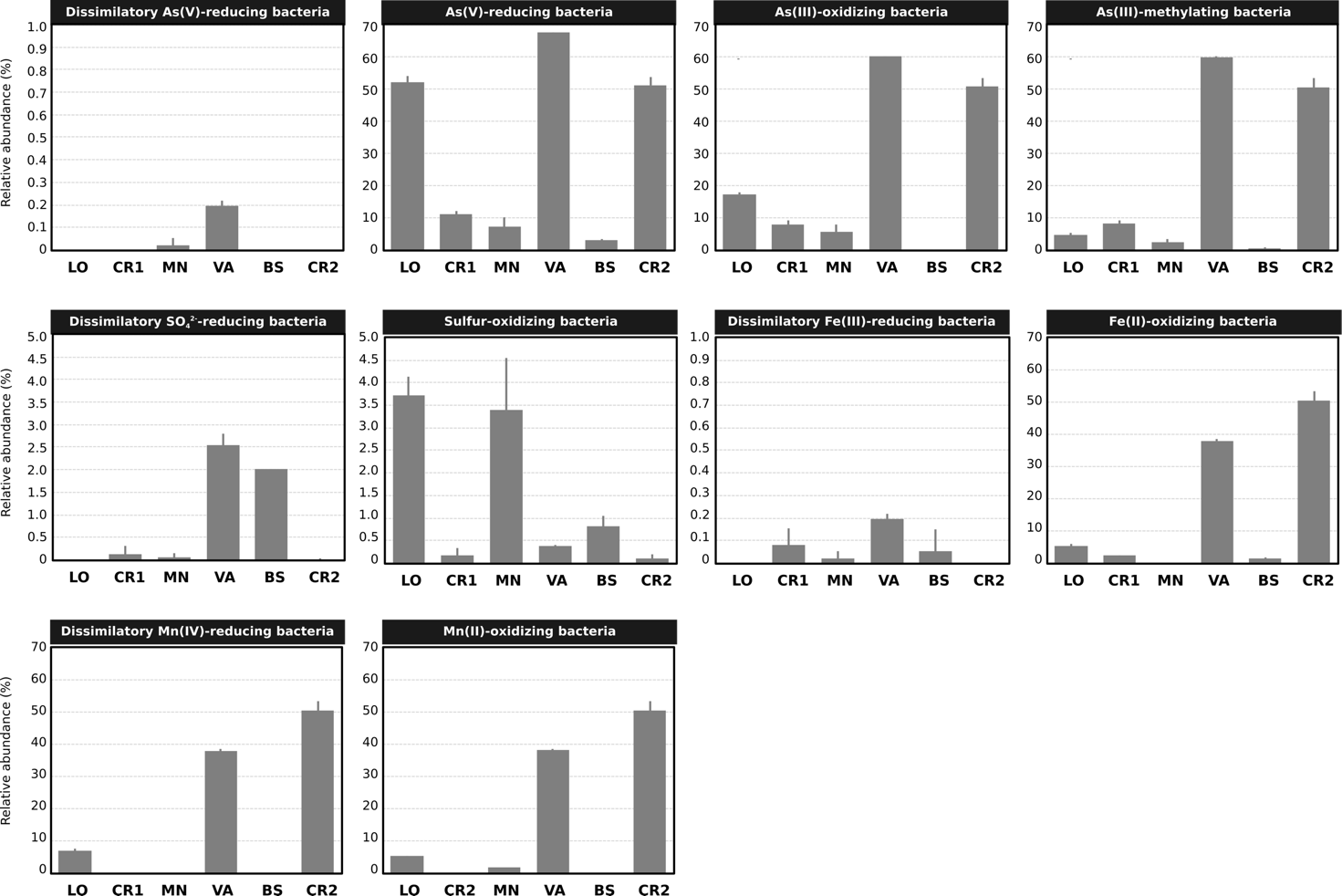


**Supplementary Figure 4.** Relative abundance of putative dissimilatory sulfate-reducing bacteria, sulfur-oxidizing bacteria, iron-reducing and -oxidizing bacteria, manganese-reducing and -oxidizing bacteria, dissimilatory arsenate-reducing bacteria, arsenate-reducing bacteria for detoxification purposes, arsenite-oxidizing bacteria and arsenite-methylating bacteria retrieved in the Illumina 16S rRNA gene library in the different sampling sites.


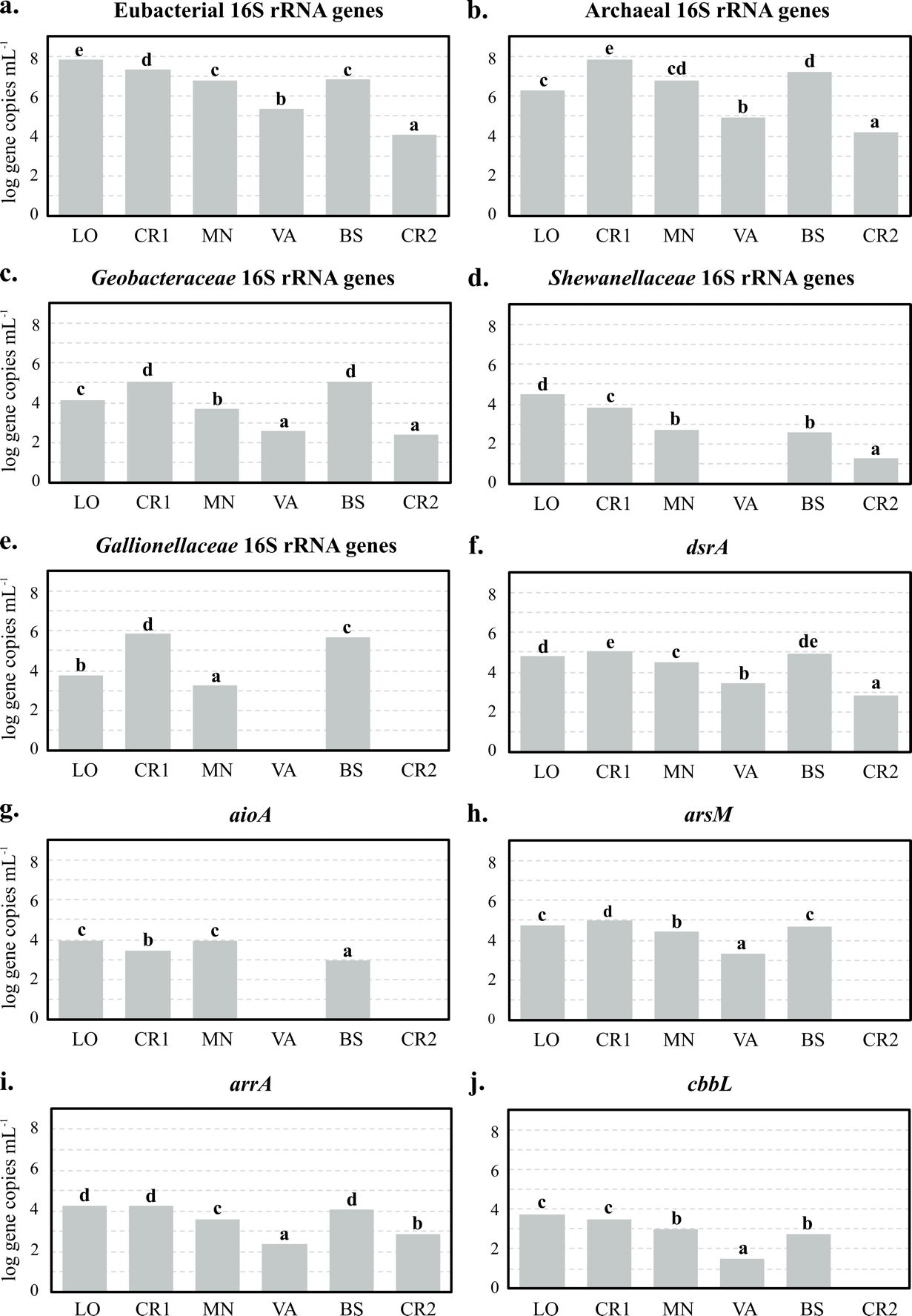


**Supplementary Figure 5**. Quantification of 16S rRNA gene copies belonging to *Eubacteria* (A), *Archaea* (B), *Geobacteraceae* (C), *Shewanellaceae* (D) and *Gallionellaceae* (E) and of genes encoding the A subunit of dissimilatory bi-sulfite reductase (*dsrA*, F), arsenite oxidase (*aioA*, G), arsenite methylase (*arsM*, H), dissimilatory arsenate reductase (I) and the A subunit of bisulfite reductase and ribulose-1,5-bisphosphate carboxylase/oxidase (*cbbL*, J). Letters indicate statistical significant groups based on ANOVA (Tukey’s test, p ≤ 0.05).

## Supplementary Tables

**Supplementary Table 1.** Chemical characteristics of sampled groundwaters.

| **ID** | **Electric conductivity** | **COD** | **Na** | **Mg** | **K** | **Ca** | **P** |
| --- | --- | --- | --- | --- | --- | --- | --- |
| Unit | µS cm^-1^ 24°C | mg L^-1^ O_2_ | mg L^-1^ | mg L^-1^ | mg L^-1^ | mg L^-1^ | mg L^-1^ |
| LO | 362 | < 15 | 10.58 ± 0.22 | 24.56 ± 0.50 | 4.17 ± 0.07 | 34.38 ± 0.36 | - |
| CR1 | 697 | < 15 | 10.58 ± 0.22 | 22.84 ± 0.29 | 5.44 ± 0.02 | 78.18 ± 0.28 | 309.21 ± 0.00 |
| MN | 459 | < 15 | 12.12 ± 0.16 | 23.46 ± 0.16 | 1.89 ± 0.0 | 58.82 ± 0.52 | 596.6 ± 225.1 |
| VA | 206 | < 15 | 6.47 ± 0.06 | 6.90 ± 0.06 | 2.50 ± 0.03 | 22.9 ± 0.22 | - |
| BS | 493 | < 15 | 12.9 ± 0.28 | 10.7 ± 0.28 | 1.51 ± 0.04 | 67.5 ± 1.28 | 0.68 ± 0.00 |
| CR2 | 553 | <15 | 8.91 ± 0.16 | 7.62 ± 0.04 | 2.03 ± 0.06 | 53.78 ± 9.48 | 154.61 ± 8.41 |

**Supplementary Table 2.** Pearson correlation of physicochemical data.

| **Parameter** | **As(V)** | **As(III)** | **Tot Fe** | **Fe(II)** | **Mn** | **NO_3_** | **NH_4_** | **SO_4_^2-^** | **T** | **Depth** | **E_h_** | **CE** | **pH** | **O_2_** |
| --- | --- | --- | --- | --- | --- | --- | --- | --- | --- | --- | --- | --- | --- | --- |
| **Tot As** | 0.57 | **0.90^*^** | 0.34 | 0.41 | 0.22 | 0.12 | 0.67 | -0.63 | -0.28 | 0.54 | -0.16 | 0.22 | -0.01 | -0.72 |
| **As(V)** |  | 0.25 | -0.40 | -0.44 | -0.47 | -0.43 | 0.56 | -0.23 | -0.24 | **0.87^*^** | 0.08 | -0.25 | 0.60 | -0.53 |
| **As(III)** |  |  | 0.36 | 0.51 | 0.34 | 0.15 | 0.43 | 0.01 | -0.48 | 0.39 | -0.21 | 0.26 | -0.07 | -0.54 |
| **Tot Fe** |  |  |  | **0.95^*^**^*^ | 0.68 | 0.70 | 0.36 | 0.50 | 0.49 | -0.47 | -0.42 | 0.72 | **-0.91^*^** | -0.36 |
| **Fe(II)** |  |  |  |  | **0.82^*^** | 0.71 | 0.23 | 0.51 | 0.26 | -0.49 | -0.25 | 0.58 | **-0.82^*^** | -0.27 |
| **Mn** |  |  |  |  |  | 0.44 | -0.25 | -0.12 | -0.04 | -0.65 | 0.28 | 0.20 | -0.48 | -0.08 |
| **NO_3_** |  |  |  |  |  |  | 0.33 | 0.84 | 0.57 | -0.50 | -0.11 | 0.16 | **-0.82^*^** | 0.25 |
| **NH_4_** |  |  |  |  |  |  |  | 0.52 | 0.38 | 0.54 | -0.54 | 0.37 | -0.29 | -0.58 |
| **SO_4_^2-^** |  |  |  |  |  |  |  |  | 0.53 | -0.11 | -0.34 | -0.03 | -0.54 | -0.2 |
| **T** |  |  |  |  |  |  |  |  |  | -0.40 | -0.36 | 0.37 | -0.71 | 0.06 |
| **Depth** |  |  |  |  |  |  |  |  |  |  | -0.21 | -0.11 | 0.60 | -0.49 |
| **E_h_** |  |  |  |  |  |  |  |  |  |  |  | **-0.84*** | **0**.48 | 0.45 |
| **CE** |  |  |  |  |  |  |  |  |  |  |  |  | -0.65 | -0.61 |
| **pH** |  |  |  |  |  |  |  |  |  |  |  |  |  | 0.03 |

*significant correlation (p < 0.05)

**significant correlation (p < 0.01)

**Supplementary Table 3.** Number of sequences produced by bacterial and archaeal 16S rRNA gene Illumina sequencing.

| **ID** | **Raw reads** | **Final reads** | **ASVs** |
| --- | --- | --- | --- |
| LO | 23516 ± 2748 | 5972 ± 949 | 94 |
| CR1 | 12722 ± 2768 | 3509 ± 519 | 85 |
| MN | 24248 ± 8390 | 11181 ± 8013 | 123 |
| VA | 29927 ± 1710 | 16526 ± 1217 | 78 |
| BS | 16667 ± 3396 | 5630 ± 1100 | 102 |
| CR2 | 24983 ± 3216 | 14166 ± 1296 | 94 |

**Supplementary Table 4.** Significantly different groups based on ANOVA (Tukey’s test, p ≤ 0.05) determined for relative abundances of phylogenetic and functional gene markers analyzed by qPCR and shown in Figure 5.

| **Target** | **LO** | **CR1** | **MN** | **VA** | **BS** | **CR2** |
| --- | --- | --- | --- | --- | --- | --- |
| *Geobacteraceae* | a | a | a | a | b | b |
| *Shewanellaceae* | a | a | a | a | a | b |
| *Gallionellaceae* | a | b | a | nd* | c | nd |
| *aioA* | a | a | b | nd | a | nd |
| *arsM* | a | a | ab | c | b | nd |
| *arrA* | a | a | a | a | a | b |
| *dsrA* | a | a | a | a | a | b |
| *cbbL* | c | c | b | a | a | a |

*nd: not determined
